# Mechanotransduction at the Plasma Membrane-Cytoskeleton Interface

**DOI:** 10.3390/ijms222111566

**Published:** 2021-10-26

**Authors:** Iván P. Uray, Karen Uray

**Affiliations:** 1 Department of Clinical Oncology, Faculty of Medicine, University of Debrecen, 4032 Debrecen, Hungary; uray.ivan@med.unideb.hu; 2 Department of Medical Chemistry, Faculty of Medicine, University of Debrecen, 4032 Debrecen, Hungary

**Keywords:** mechanotransduction, plasma membrane, cytoskeleton, cancer, gastrointestinal

## Abstract

Mechanical cues are crucial for survival, adaptation, and normal homeostasis in virtually every cell type. The transduction of mechanical messages into intracellular biochemical messages is termed mechanotransduction. While significant advances in biochemical signaling have been made in the last few decades, the role of mechanotransduction in physiological and pathological processes has been largely overlooked until recently. In this review, the role of interactions between the cytoskeleton and cell-cell/cell-matrix adhesions in transducing mechanical signals is discussed. In addition, mechanosensors that reside in the cell membrane and the transduction of mechanical signals to the nucleus are discussed. Finally, we describe two examples in which mechanotransduction plays a significant role in normal physiology and disease development. The first example is the role of mechanotransduction in the proliferation and metastasis of cancerous cells. In this system, the role of mechanotransduction in cellular processes, including proliferation, differentiation, and motility, is described. In the second example, the role of mechanotransduction in a mechanically active organ, the gastrointestinal tract, is described. In the gut, mechanotransduction contributes to normal physiology and the development of motility disorders.

## 1. Introduction

Organisms necessarily respond to their environment for survival and adaptation. At the cellular level, cells respond not only to chemical messages, such as hormones, but also to mechanical messages. The transduction of mechanical messages into intracellular biochemical messages is termed mechanotransduction and is necessary for an organism/cell to respond adequately to its environment. Virtually every cell type responds to mechanical cues for survival, adaptation, and normal homeostasis. Mechanical messages can include shear stress, pressure, stiffness/compliance, or stretch. Just as the localization, concentration, and timing of biochemical messages modulate their effects, the magnitude, direction, and spatial and temporal aspects of mechanical signals modulate their effects [1]. Mechanical cues from the extracellular matrix, cell membrane, and cytoskeleton all participate in two-way communication with biochemical signaling within the cell. On one hand, mechanical stimuli result in intracellular biochemical responses altering signaling pathways that can extend even to the nucleus, where mechanical signals can induce gene expression changes [2]. On the other hand, intracellular biochemical signals modulate mechanical signals via altered extracellular matrix, cytoskeletal proteins, and cell membrane proteins. In addition, cells are capable of generating force and, thus, modulating the mechanical signaling of surrounding cells and the extracellular matrix. Thus, physical forces in the cellular cortex and plasma membrane and intracellular biochemical signaling cooperate in complex two-way communication to sense and adapt to mechanical signals [3,4].

Significant advances in our understanding of molecular signaling pathways have been made in the last several decades. However, the contributions of mechanotransduction to cellular responses are poorly understood and often overlooked. Mechanotransduction plays crucial roles in a wide variety of cellular processes and disturbances in these processes lead to disease development. Furthermore, cellular responses to drug treatments can be affected by the biomechanical properties of tissues [5]. Therefore, understanding the role of mechanotransduction in cellular processes is important in understanding disease development.

In this review, we will discuss basic cellular biomechanics and the role of interactions between the cytoskeleton and cell-cell/cell-matrix adhesions in transducing mechanical signals in mammalian cells. In addition, we will discuss mechanosensors that reside in the cell membrane. Finally, we will describe examples of two systems where mechanotransduction plays a significant role in disease development. The first is the role of mechanical signals in cancerous cells. In this system, the role of mechanotransduction in cellular processes, including proliferation, differentiation, and motility, is described. In the second example, the role of mechanotransduction in a mechanically active organ, the gastrointestinal tract, is described. In the gut, mechanotransduction contributes to normal physiology and the development of motility disorders.

## 2. An Overview of Cellular Biomechanics

Mechanical signaling is involved in virtually all physiological processes. Cells are required to sense a wide variety of mechanical signals, which are defined by the magnitude, direction, and spatial and temporal variations of the signals. Moreover, cellular responses to the variety of mechanical signals are distinct, depending on the cell type, composition, and conditions, i.e., different cell types may respond differently to the same signal and the same cell may respond differently depending on the conditions and intracellular regulatory signaling pathways active at any one moment.

Cellular responses to mechanical signals are dependent on the viscoelastic properties of cells, which are largely dependent on the highly dynamic cytoskeleton [6,7]. The viscoelastic properties of the cytoskeleton arise from the properties and dynamic interactions of actin, microtubules, and intermediate fibers [8]. Intermediate filaments are the most elastic (i.e., the least stiff) and the microtubules are the stiffest component of the cytoskeleton. These three cytoskeletal elements self-organize into heterogeneous highly dynamic networks and bundles with the help of cross-linkers and motor proteins; the mechanical properties of this combined network lies between rigid rods and highly flexible coils [9]. The ability of the cell to sustain and respond to mechanical stress is dependent, not on the individual filament properties, but on the properties of the complex cytoskeletal network, which is constantly adapting in response to both chemical and mechanical cues in the cell’s environment [10]. The cytoskeleton can generate tension and transmit tension throughout the cell, including the nucleus. Unlike simple polymers like polyacrylamide, this complex cytoskeleton becomes stiffer in response to deformation [9]. Moreover, many mechanosensors, such as mechanosensitive ion channels, reside on or in association with the cell membrane. Transmission of cellular stress to the fluid membrane is dependent on the coupling of the cell membrane with the cytoskeleton, at cell-cell or cell-matrix adhesions [11]. Interaction of the cytoskeleton with cell-cell and cell-matrix adhesions is necessary for sensing, transmitting, and responding to mechanical signals.

## 3. Role of the Cytoskeleton in Mechanotransduction

### 3.1. Microtubules

Microtubules are the stiffest of the three cytoskeletal components [12]. Microtubules can span the length of a eukaryotic cell and can withstand high compressive loads to maintain cell shape [13]. Microtubules can switch rapidly between stably growing and rapidly shrinking processes to reorganize quickly [14]. Microtubules consist of tubulin heterodimers organized into cylindrical structures, and the organization and dynamics are significantly influenced by tubulin isotypes [15]. The role of microtubules in mechanotransduction is not well understood; however, a few studies highlight the importance of the microtubule network in mechanotransduction. Rafiq et al. showed that microtubules modify both focal adhesions and podosomes via KANK proteins to regulate the actomyosin cytoskeleton [16]. In a breast cancer model, matrix stiffening promoted glutamylation of microtubules to affect their mechanical stability [17]. Joca et al. showed that increased stretching of cardiomyocytes induced microtubule-dependent changes in NADPH oxidase and reactive oxygen species [18]. Mechanical stimulation of Chinese hamster ovary cells induced rapid depolymerization of microtubules at the indentation point and slow polymerization of microtubules around the perimeter of the indentation point [19]. Tension stabilizes microtubule coupling with kinetochores in yeast [20]. Overall, these studies show that microtubules can sense and respond to mechanical cues to participate in mechanotransduction.

### 3.2. Intermediate Filaments

Intermediate filaments are shorter than microtubules and actin fibers, are highly flexible and extensible, and exhibit strain-induced strengthening [21,22]. These properties of intermediate filaments make them sensitive to mechanical stress and convey mechanical resistance to cells [22,23]. Like the other cytoskeletal components, the formation of intermediate fibers is regulated in a cell- and context-dependent manner [24]. Intermediate filaments are assembled from a group of well-conserved proteins that share a common structure: a central a-helical domain flanked by two variable non-helical domains, which account for the functional diversity of intermediate fibers [24]. Like the other two cytoskeletal components, intermediate filament assembly is dynamic. Interestingly, the precursor pools are detected mostly at the periphery or protrusions of cells [25].

Intermediate fibers interact with cell-cell and cell-matrix adhesions [24]. Due to their elasticity, intermediate fibers transmit mechanical signals via cell-surface adhesions to the intracellular space and neighboring cells to control cell stiffness. For instance, fluid shear stress induces rapid reorganization of intermediate fibers in endothelial cells [26]. Mechanical stress induces phosphorylation of the regulatory heads of intermediate fiber proteins to regulate intermediate fiber reorganization and cell stiffness in epithelial cells [27,28]. Thus, intermediate fibers play a crucial role in sensing and transducing mechanical signals.

### 3.3. Actin Filaments

Actin filaments not only transmit force through the cell but can also generate force through polymerization [29]. The non-covalent polymerization of actin supports a variety of non-muscle cell movements, such as cell migration and division [30]. The basic building blocks of actin filaments are the actin monomers, which assemble into double-stranded helices [31]. Therefore, actin exists in two pools, filamentous actin (F-actin) and free actin, referred to as globular actin (G-actin). Actin filaments are semiflexible and dynamic, enabling cells to rapidly change shape and respond to intracellular and extracellular forces [10]. Actin filaments are semi-flexible on the scale of the cell length (10 μm). Therefore, shorter filaments behave as rigid rods and longer filaments can bend [32]. Actin filament bending is accompanied by twisting due to the helical structure of actin filaments [33]. Actin filaments sometimes form bundles that can withstand higher compression forces [34]. Changes in actin fiber tension are transmitted across the cell and to cell-cell and cell-matrix adhesions. Actin cross-linking proteins play an important role in the formation of actin networks and bundles and, thus, play an important role in the mechanical properties of cells.

**Figure 1:**
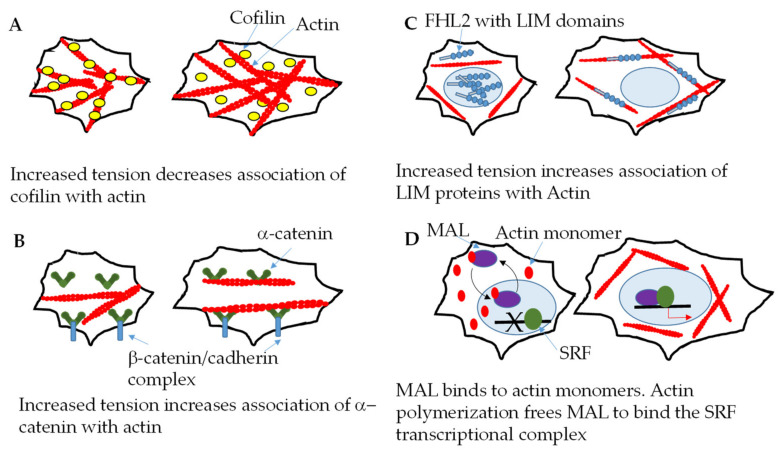
Examples of mechanosensing by actin filaments. (**A**) Increased tension of actin filaments decreases cofilin association, resulting in delayed actin filament severing. (**B**) Increased actin filament tension, within the cellular range, results in increase α-catenin binding either at the cell cortex or intracellularly. (**C**) Increased actin filament tension increases the binding of FHL2-containing proteins, excluding these proteins form the nucleus. (**D**) MAL binds to actin monomers in both the cytoplasm and the nucleus. Stimulation of cells with serum increases actin polymerization and decreases the availability of actin monomers. MAL then becomes available to bind to the SRF complex.

As mentioned previously, the actin network is highly dynamic, and the actin cytoskeleton is highly responsive to mechanical cues. Several examples of mechanosensing by actin filaments are shown in Figure 1. One mechanism by which actin filaments sense tension is through altered binding to other proteins in response to altered tension/force. Hayakawa et al. showed that the cofilin binding rate decreased and actin severing was delayed when the tension of single actin filaments was increased using optical tweezers (Figure 1A) [35]. Mei et al. showed that increased tension of actin filaments increased α-catenin binding (Figure 1B) [36]. Hosseini et al. showed that increased tension increases binding of the actin cross-linker, α-actinin-4, to actin [37]. LIM domain proteins, including members of the zyxin, paxillin, and FHL families, accumulate on mechanically stimulated actin via their LIM domains; increased binding of FHL prevents nuclear localization (Figure 1C) [38].

Interestingly, changes in F-actin regulate the Hippo signaling pathway. The mammalian Hippo pathway, which plays a key role in cellular differentiation and proliferation responses to mechanical signaling, consists of a kinase cascade of the mammalian sterile 20-like kinase (MST)1/2 and large tumor suppressor (LATS)1/2 and an adaptor protein (SAV1). When phosphorylated, MST1/2 and LATS1/2 prevent yes-associated protein (YAP)/transcriptional coactivator with PDZ-binding motif (TAZ) (YAP and TAZ have overlapping redundant functions) from entering the nucleus and activating genes regulating cell survival and proliferation; phosphorylated YAP is retained in the cytoplasm and degrades [39]. Increases in F-actin induce nuclear translocation of YAP. F-actin may also modulate YAP activity through Hippo-independent pathways. Actin polymerization affects transcriptional regulation by serum response factor (SRF) signaling. MAL, a SRF coactivator, binds to nuclear actin monomers, which prevents MAL interaction with the SRF transcriptional complex. When cells are stimulated with serum, increased actin polymerization decreases the availability of actin monomers and MAL binds to the SRF complex (Figure 1D) [40].

### 3.4. Cell Cortex

The cytoskeleton underlying the plasma membrane, called the cell cortex, plays an important role in mechanotransduction. The specialized cytoskeleton of the cell cortex is the interface between the cytoskeleton and the plasma membrane and regulates not only cell shape, but also plasma membrane organization [41]. Like other parts of the cell, the cytoskeleton at the cell cortex is dynamic, allowing it to sense and respond to both biochemical and mechanical signals. The plasma membrane interacts with cytoskeletal actin at the cell cortex in a mechanosensitive manner through a variety of binding motifs, including the α-actinin [42] and calponin homology binding domains [43], and/or linker proteins, such as ezrin, radixin, moesin, and filamin A [44,45,46]. The ERM proteins (ezrin, radixin, and moesin) contain amino-terminal FERM domains, which interact with the cell membrane, and carboxyterminal F-actin-binding domains [47]. ERM proteins participate in crosstalk between mechanosensitive plasma membrane proteins, such as TREK1 and TRPV6, and the actin cytoskeleton [48,49]. Filamin A binds to actin at the N-terminal and interacts with a variety of membrane and submembrane proteins, such as integrins and FilGAP, via cryptic sites that change depending on the mechanical deformation [50].

Cortical actin below the plasma membrane surface plays a significant role in organizing membrane proteins and participates in mechanotransduction. Gawrishankar et al. demonstrated that short dynamic actin filaments interact with plasma membrane proteins containing actin-binding motifs to organize nanoclusters [51]. Membrane tension influences cortical actin and vice versa [52]. Alterations in ERM proteins or filamin A both alter membrane tension [53,54].

Interestingly, force can be directly and rapidly transmitted from the cell cortex to the nucleus, resulting in epigenetic or transcriptional changes. A mechanical link between the cell membrane and the nucleus was demonstrated by Maniotis et al. [55]. This mechanical link requires integrin, actin, intermediate filaments, and microtubules [56]. The nuclear Linker of Nucleoskeleton and Cytoskeleton (LINC) complex consists of nesprins in the outer nuclear membrane and SUN proteins in the inner nuclear membrane. The LINC complex responds to mechanical changes in the extracellular matrix via integrins and cell-cell contacts via cadherins [57,58].

## 4. Role of Cell Membrane Proteins in Mechanotransduction

The mammalian cell membrane is easily deformed by mechanical forces and mechanosensitive proteins in the cell membrane are crucial players in mechanotransduction. Proteins residing in the cell membrane are subjected to local changes in force and transduce these mechanical cues into changes in intracellular signaling. The most well-known and widely studied mechanosensors are mechanosensitive ion channels; however, GPCRs and other mechanosensitive proteins have also been discovered recently.

### 4.1. Ion Channels

Mechanosensitive ion channels are expressed in a wide variety of cell types in virtually every physiological system. Mechanosensitive channels, by definition, span the plasma membrane and are directly activated by mechanical stress; the mechanical stress is converted to an electrochemical signal by ion channels and eventually leads to changes in downstream signaling. The mechanism by which mechanosensitive ion channels are activated is poorly understood for the most part; however, tethering to the extracellular matrix or the intracellular cytoskeleton, the direct effects of plasma membrane expansion, and/or interactions with lipid rafts or other membrane lipid domains may play a role. Many ion channels are mechanosensitive in eukaryotic cells [59]. We will briefly discuss epithelial sodium channel (ENaC), Piezo, TREK, transient receptor potential (TRP), and big potassium (BK) channel families; however, other ion channels also contribute to mechanotransduction. Table 1 shows the diverse roles of mechanosensitive ion channels in mammals. Of note, as techniques become more sophisticated, mechanisms for the mechanosensitivity of channels are being elucidated, and some studies conflict with earlier work concerning the mechanosensitivity and role(s) of ion channels.

#### 4.1.1. ENaC Superfamily

Members of the ENaC superfamily of ion channels form homotrimers or heterotrimers with two transmembrane regions per subunit [94]. Although the specific mechanism for mechanical gating of this family of ion channels is not well understood, the extracellular loop appears to be sensitive to shear stress. A recent study showed that ENaC activity in human pulmonary microvascular endothelial cells was increased in response to shear stress and the extracellular loop appears to act as a tether to the extracellular matrix [95]. ENaC responds to shear stress in both conduit and resistance arteries and changes in ENaC activity in response to shear stress alter intracellular endothelial nitric oxide synthase activity to regulate vasoconstriction [96].

#### 4.1.2. Piezo Channels

The recently discovered Piezo channels are trimeric proteins with a large number (24–40) of transmembrane regions [97,98]; two Piezo channels (Piezo1 and Piezo2) exist in vertebrates [99]. The mechanosensitivity of these complex channels is poorly understood; however, mechanosensitivity may be conveyed via interaction with regulator proteins or changes in conformation. Notably, the membrane tension required to gate Piezo1 channels is within the physiological range and mechanical manipulation was enough to activate the channels in an experimental lipid bilayer model [100,101]. Interactions of Piezo channels with the extracellular matrix and cytoskeleton modulate the mechanosensitivity of Piezo channels. Piezo channels are 10x more sensitive to membrane tension when tethered to the extracellular matrix. The presence of collagen IV, in particular, sensitized Piezo channels to mechanoactivation [102]. Piezo channels are bound to the actin cytoskeleton by the E-cadherin complex, and the absence of E-cadherin or β-catenin desensitizes Piezo1 channels [103]. In contrast, removal of filamin A activates Piezo channels, suggesting that interaction with the cytoskeleton through filamin A can desensitize Piezo channels [104]. Piezo channels transduce mechanical forces in a wide range of physiological processes that require exquisite control. A combination of extracellular (via interactions with the extracellular matrix), intracellular (via interactions with the cytoskeleton), and cell membrane (membrane stiffness) forces likely contribute to the activity of Piezo channels.

In the skin, Piezo channels play a significant role in touch sensitivity [73]. In endothelial cells, Piezo channels respond to both shear stress and stretch. Depletion of Piezo1 reduces endothelial nitric oxide activity in endothelial cells indicating that Piezo channels can regulate vasoconstriction [68]. In addition, activation of Piezo1 channels by shear stress induces ATP release from endothelial cells [72]. The subcellular location of Piezo1 changes to the leading apical lamellipodia in response to shear stress, indicating that Piezo channels play a role in cell migration [68,70]. Stretch-activation of Piezo1 plays a role in sensing flow and bladder extension in the urinary tract [69]. In osteoblasts, Piezo 1 channels are down-regulated in response to microgravity and may play a role in altered bone growth during microgravity [71]. Overall, Piezo channels play a significant role in a wide variety of cell types.

#### 4.1.3. TREK Channels

The TREK channel family consists of two-pore selective potassium channels (TREK1, TREK2, and TRAAK), which are widely expressed in the central and peripheral nervous systems [105]. Several members of this family can be activated by mechanical signals, including stretch and cell swelling [106]. The TREK-1 channel is directly responsive to membrane tension [107]. The mechanism underlying mechanoactivation of these channels may involve exposure of an increased cross-sectional area of the channel during membrane stretch that alters interactions with the lipid bilayer; altered interactions with the lipid bilayer support conformational changes that favor pore opening [108,109,110]. Interaction of TREK-1 with the actin cytoskeleton may modulate the mechanosensitivity of the channel [48]. Knockdown of TREK or TRAAK channels causes hypersensitivity to mechanical stimuli [109]. In addition, TREK channels may be involved in the development of arrhythmias and remodeling in the heart [111,112].

#### 4.1.4. TRP Channels

TRP channels are a large family of nonselective cation channels with a tetrameric structure containing 6 transmembrane domains [113]. The direct activation of TRP channels by membrane tension is controversial [114,115]. Force sensing and transduction may be mediated by the interactions of the TRPP1/TRPP2 complex with the extracellular matrix, focal adhesions, the cytoskeleton, or other mechanosensitive channels like Piezo1 [116,117]. Mechanotransduction of TRP channels plays important roles in cardiovascular homeostasis, nociception, renal function, and neural function [117]. Shear stress activates transient calcium release at the leading edge of migrating fibroblasts via TRPM7 [118]. TRPP’/TRPP2, TRPV4, and TRPC1 all modulate vascular smooth muscle contractility in response to various mechanical signals [84,119,120,121].

#### 4.1.5. BK Channels

BK channels are cytosolic Ca^2+^-activated potassium channels, consisting of tetramers of α and β subunits [122]. The functional diversity of BK channels is conveyed by the expression of different α/β subunits and splicing variants [123,124]. BK channels contain a stress-axis regulated (STREX) domain at the C-terminus, which can be activated by stretching the cell membrane [125]. Other domains also play a role in the stretch activation of BK channels, as demonstrated by stretch activation of BK channels lacking the STREX domain in colonic smooth muscle [92]. BK channels are expressed predominantly in the smooth muscle of various organs and the brain and pancreas and play a significant role in neuronal excitability, hormone secretion, and smooth muscle contractility [126].

### 4.2. G-Protein Coupled Receptors

G-protein coupled receptors (GPCRs) are a well-known family of 7-transmembrane-domain receptors. Much is known about ligand activation of GPCRs and the downstream signaling pathways associated with GPCR activation. Recent research suggests that mechanical stimuli can also activate a number of GPCRs in the absence of their relevant agonists, resulting in translocation of their corresponding G proteins [127]. Early evidence supporting mechanosensitive GPCRs came from the angiotensin II type I (AT1) receptor. Komuro et al. showed that mechanical stretch induced the association of the AT1 receptor with janus kinase 2 and the translocation of G proteins to the cytosol [128]. Furthermore, increased stretch induces cardiac hypertrophy in vivo in the absence of angiotensin II [128]. Subsequent studies showed that hypotonic swelling of the cell membrane resulted in agonist-independent recruitment of β-arrestin to the AT1 receptor to the same degree as maximum agonist stimulation [129]. The authors of this study went on to show that other GPCSs, including the H1 histamine receptor and the muscarinic receptor (M5R), could also be activated by stretch in the absence of ligands, indicating that the G_q/11-_coupled receptors share a common mechanosensitive activation mechanism [129]. The proposed mechanism for stretch-induced activation of GPCRs is conformational change. This mechanism is supported by bioluminescence resonance energy transfer and fluorescent resonance energy transfer studies [129,130]. In addition to G_q/11-_coupled receptors, G_i/o-_coupled receptors may be activated in the same ligand-independent manner [131]. Interestingly, the inverse agonist, candesartan, prevents stretch-activation of the AT1 receptor, probably by locking the receptor in an inactive conformation [128]. Stretch activation of GPCRs plays key roles in the development of cardiac hypertrophy and myogenic vasoconstriction.

## 5. Role of Cell-Cell Adhesions in Mechanotransduction

Cell-cell junctions are specialized regions of the plasma membrane consisting of protein complexes that couple adjacent cells. Cell adhesions allow tissues to resist external and internal forces and allow sensing and transmission of force between cells. Cell-cell adhesions are unique to the cell type, tissue type, developmental stage, and physiological/pathological conditions, and may possess different mechanical properties, i.e., mesenchymal tissue cell-cell adhesions may have different tensile strengths than epithelial cell contacts [132]. The extracellular domains of transmembrane receptors within the cell-cell junctions interact with adjacent cells while the intracellular domains interact with signaling complexes and the cytoskeleton. Communication between the cell-cell junctions and the cytoskeleton are two-way and both intrinsic and extrinsic forces affect mechanotransduction at the cell-cell junctions. Forces at the cell-cell junctions can directly impact cellular processes, such as proliferation and differentiation. As indicated above, the Hippo pathway plays a pivotal role in mechanotransduction processes. In addition, significant cross-talk between Wnt signaling and cell-cell adhesions impacts cell differentiation and migration.

Adherens junctions are coupled to the cytoskeleton through cadherin complexes. Cadherins, a family of transmembrane proteins, are under tension at cell junctions from both internal and external sources and can transmit tension both ways [133,134]. The intracellular domain of cadherins is bound to β-catenin, which is bound to α-catenin [135]. In the cadherin complex, α-catenin may be a mechanosensor [132,136,137]; α-catenin binds to actin filaments in a tension-sensitive manner and the α1-helix of α-catenin is a mechanosensing motif that enhances binding to actin when exposed [138]. While vinculin has been more widely studied in integrin-based focal adhesions, vinculin can also act as a mechanosensor in adherens junctions; tension transmitted via VE-cadherin in endothelial cells unfolds α-catenin and reveals binding sites for vinculin [139]. Recruitment of vinculin to the adherens junction stabilizes α-catenin [140]. Many other potential mechanosensitive proteins surround the adherens junctions in the cortical area of the cells and in communication with the cytoskeleton, including myosin motors [132]. Myosin motor proteins accumulate at focal adhesions in response to mechanical signaling, leading to changes in downstream signaling pathways [132,141]. For instance, non–muscle myosin IIA negatively regulates the accumulation of the Rac GEF, β-Pix, in focal adhesions [142].

The cell-cell junctions of epithelial and endothelial monolayers are referred to as tight junctions because they limit the passage of ions and solutes through the monolayer. Tight junctions are also multimeric protein complexes and include the transmembrane proteins, claudins, occludin, junctional adhesion molecules (JAMs), and intracellular proteins, zona occludins (ZO), MAGI, MUPP1, and PATJ [143]. ZOs and cingulin proteins anchor tight junctions to the actin cytoskeleton [144] and alterations in ZO1/2 in epithelial and endothelial cells alters actomyosin cytoskeletal tension [145,146,147]. Shear stress downregulates occludin and claudin expression and increases vascular permeability [148]. JAMs regulate cell motility in a taxol-dependent manner, indicating that microtubules are involved [149].

Desmosomes are cell-cell contacts in tissues, such as the myocardium, bladder, and skin, that experience mechanical stress [150]. Unlike adherens junctions and tight junctions, desmosomes couple with intermediate filaments. Two cadherin subtypes, desmogleins and desmocolllins, make up the transmembrane component of desmosomes. The intracellular domains of these cadherins bind to plakoglobin and plakophilins and desmosomes are linked with intermediate filaments via desmoplakin [143]. Interestingly, using a FRET-based tension sensor system, Price et al. showed that cytoskeleton-generated forces have little impact on desmoplakin tension, but external forces impact desmosome tension [151]. The authors suggested that desmosomes are specialized for stress absorption. In conflict with Price et al., using a force-sensing desmoglein-2 construct, Baddam et al. showed that desmoglein experiences low-level tension in non-contracting cells [152]. The reasons for this discrepancy are unclear, but investigations into the role of desmosomes in mechanotransduction are in the early stages. Desmosomes interact with adherens junctions and may affect mechanotransduction in this way.

## 6. Role of Cell-Matrix Adhesions in Mechanotransduction

Focal adhesions are large protein complexes that consist of integrins and a large array of adaptor proteins. Focal adhesions begin as nascent adhesions with only a few integrins. While some nascent adhesions are short-lived, other nascent adhesions mature into stable focal adhesions, depending on intracellular and extracellular conditions [153]. Focal adhesions mechanically connect the extracellular matrix to the cytoskeleton via stress fibers, and communication between the extracellular matrix and intracellular proteins via integrins is two-way. Cell-matrix adhesions are mechanosensitive structures that grow and shrink in response to mechanical signals. For example, on rigid substrates, focal adhesions mediate actin filament growth via Rho signaling [154]. Contraction of the cytoskeleton is transmitted to the extracellular matrix to promote changes, such as fibronectin fibrillogenesis [155]

Integrins are composed of α and β subunits and 26 different integrins are expressed in mammals [156]. Signaling at focal adhesions is diverse and complex. The diversity of mechanotransduction through focal adhesions depends on the integrin makeup of the focal adhesions, trafficking of integrins to focal adhesions, the properties of the extracellular matrix, and the intracellular signaling complexes associated with the focal adhesions [157]. The α/β integrin subunits assemble in different combinations, resulting in different substrate (extracellular matrix components) affinities and different intracellular signaling [156,158]. Integrins act as receptors for extracellular proteins, including collagen, laminin, fibronectin, vitronectin, and thrombospondins [158]. The viscoelastic properties of the extracellular matrix also affect the transduction of force through the integrin complex. Thus, the composition of the extracellular matrix affects mechanotransduction through focal adhesions. Integrin trafficking to and from the cell membrane is regulated by endocytosis, which is regulated by a number of different cell- and context-dependent signaling pathways [159]. In addition, integrins associate with different intracellular signaling pathways, including the small GTPases, RhoA and Rac, the Hippo signaling pathway (discussed above), and focal adhesion kinase and Src. Thus, both intracellular and extracellular factors, along with the molecular makeup of the integrin complex itself, affect the transduction of force through focal adhesions.

Integrins couple to the cytoskeleton through F-actin binding proteins, such as talin and vinculin. Talin is a mechanosensitive protein; when talin is stretched, cryptic sites for vinculin binding are exposed [160,161]. Vinculin is a component of both focal adhesions and adherens junctions and binds to talin and α- and β-catenin, among other binding partners [162]. Vinculin binding stabilizes focal adhesions by locking talin in the active conformation and modulating talin binding to actin [163]. Two isoforms of talin with different mechanotransductive properties are expressed in mammals; talin-1 is widely expressed whereas talin-2 is expressed predominantly in the heart, skeletal muscle, and brain [164]. Talin forms a “molecular clutch” that transmits force generated by actin polymerization to the cell membrane for cell motility [165]. Thus, talin both senses and transmits force. Talin-vinculin interactions play a significant role in cell migration regulated by intracellular tension.

Another stretch-sensitive protein, p130Cas, is also localized to focal adhesions. Both the SH3 domain and Src-binding domains are required for Cas localization to focal adhesions [166]. Cell stretching and mechanical extension of p130Cas result in increased phosphorylation by Src-family kinases [167]. The increased phosphorylation of p130Cas in response to stretch was not dependent on increased Src kinase activity. Thus, the increased phosphorylation of p130Cas is likely due to the increased susceptibility of the extended protein to phosphorylation.

Integrins also connect to actin filaments via other protein complexes, such as kinlin, PINCH, and the parvin complex [168]. Integrin-linked kinase interacts with PINCH and parvin to form a complex linking integrins with actin filaments [168] to modulate many cellular functions, including cell spreading, fibronectin deposition, and cell proliferation [168].

## 7. Mechanotransduction in Cancer

In contrast to the reliance of tissues on mechanotransduction for homeostatic regulation, mechanotransduction is part of the disease process in cancer. The mechanotransductive processes fall into two main categories, ‘cell autonomous’ responses and intercellular communication between cancer cells and their microenvironment. Cancer cells experience increased pressure either through solid stress due to increased cell mass, as tumors are restricted to a confined space defined by preexisting stroma or neighboring organs, or through elevated interstitial fluid pressure by edema development. Increased stiffness and altered tissue properties, as well as altered tissue microarchitecture, arise from and act through factors adjacent to the tumor cells, and usually occur as part of the disease-specific interaction with mesenchymal elements. On the one hand, these factors fundamentally change cancer cell behavior and disease progression. On the other hand, new tissue functions may be introduced by these processes, such as stemness, epithelial plasticity, and therapeutic resistance.

### 7.1. Determinants of Stiffness and Tissue Microarchitecture

**Figure 2:**
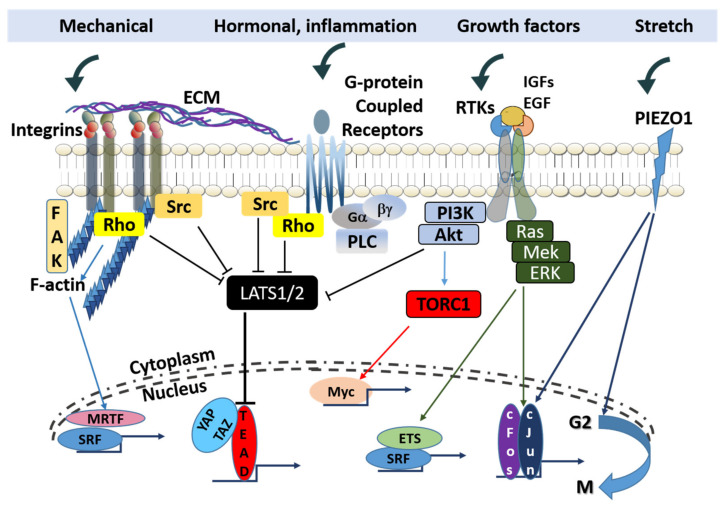
The integration of mechanical, hormonal, and growth signaling determining transcriptional regulation. Signaling routes in cancer cells activated by mechanical forces and stretching converge with growth pathways mediated through receptor tyrosine kinase (RTK) growth factor receptors and G-protein coupled receptors (GPCRs) upon LATS1/2, to modulate the Hippo pathway. Shared targets of the F-actin kinase (FAK) and Piezo1 include the serum response factor (SRF) and AP-1. (blunt ends indicate inhibition and arrows indicate simulation).

Positional control over the activity of growth factor and cytokine receptors to receive signals from and export signals to the surrounding mesenchymal tissue is primarily exerted through distinct members (not all members) of the integrin family. Select integrin isoforms influence the localization of specific cancer subtypes or the site of metastatic invasion. The integrin complex ανβ3 combined with the PDGF-β receptor is associated with enhanced proliferative signaling in pediatric glioblastoma [169], and integrin β1 mediates activation of focal adhesion kinase (FAK) for metastatic dissemination of cancer cells to the lungs [170]. Integrin functions include the activation of intracellular signaling and organization of the cytoskeleton, thereby affecting a number of cell fate transitions. Upon ligand binding, integrins cluster and engage cytoskeletal linker proteins to regulate the cellular actin network. Integrins also activate FAK or Src family kinases to transmit pro-mitogenic and pro-survival pathways (Figure 2).

Once carcinoma cells acquire invasive properties and induce changes in the extracellular matrix (ECM), various host cells (fibroblasts, macrophages, endothelial cells, and immune cells) are recruited to promote the survival of the carcinoma cells [171]. Subsequently, the ECM is jointly produced by these host cells in a concerted manner, undergoing significant changes in structure, composition, and mechanical characteristics [172]. Altered mechanical properties derive partly from the more linearized and crosslinked nature of collagen I at the tumor-stroma interface, as a result of HIF1α-induced elevated lysyl oxidase (LOX) activity [173]. In addition to increasing the rigidity of the tumor matrix, these changes up-regulate integrin signaling and stimulate cancer cell proliferation.

Integrins by themselves do not transform cells. Instead, the ability of certain integrin heterodimers, i.e., ανβ3 and α6β4, to positively regulate tumorigenesis is based on their propensity to signal through Src, c-Met, EGFR, Her-2, and other oncogenic RTKs [174,175]. Another collagen-binding integrin complex, α10β1, supports tumorigenesis through RICTOR and TRIO to activate Rac/P21-activated kinase and mTOR in myxofibrosarcoma [176].

Another class of targets for tyrosine phosphorylation mediated by integrins is the caveolin proteins of cell membrane microdomains in lipid rafts. Phosphorylation of these targets leads to the activation of Rho kinases, allowing cancer-associated fibroblasts (CAFs) to exert mechanical force. The resulting changes render the tumor stroma favorable for cell migration and metastasis [177]. Alternatively, a classic way of activating CAFs is through the action of transforming growth factor-β (TGF-β), resulting in myofibroblasts that exert significant contractile forces [178].

In addition to influencing the progression of cancer, the stromal composition of premalignant tissue has a significant impact on the factors predisposing epithelial cells to undergo transformation towards a cancerous phenotype. Mammographic density and fibrous stroma density are strong risk factors for mammary carcinomas [179,180]. Downstream effectors include activation of JNK1 stress signaling, increased COX2 expression, inhibition of TGFβ signaling, and correlation with Syndecan-1 expression in breast cells [181,182,183]. Notably, syndecan-1 may play a role in mechanosensing through modulation of Rho-associated signaling pathways, the nuclear localization of YAP/TAZ, and SMAD2/3 phosphorylation [184]. In conclusion, factors that dictate mesenchymal structure and the signaling pathways used by proliferating cells to monitor mechanical cues may offer viable preventive and therapeutic targets in the future.

### 7.2. Transcriptional Footprint of Mechanical Cues in Cancer Cells

Multiple mechanisms are utilized by cancer cells for the transduction of mechanical signals to the nucleus. Activation of the serum response factor (SRF) is primarily responsive to Rho kinase signaling through the actin-dependent translocation of the myocardin-related transcription factor (MRTF) MAL. Actin polymerization was shown to release cytoplasmic MAL, allowing it to translocate to the nucleus and activate SRF [40]. In the absence of Rho activity, filamentous actin converts to monomeric G-actin, which triggers actin-dependent nuclear export of MAL and the silencing of SRF-induced transcription [185]. Therapeutically, the pharmacological blockade of the TRPM7 cation channel may utilize MRTF-dependent transcriptional control to inhibit carcinogenic activity. A novel negative gating modulator, NS8593, was recently shown to inhibit Mg^2+^-influx and the phosphorylation of Rho kinase, leading to transcriptional inactivation of MAL and senescence of hepatocellular carcinoma cells [186].

An intriguing connection between cell density and the cell cycle was demonstrated by Gudipaty et al., who investigated the factors that control the balance between cell division and cell attrition. Moderate mechanical stretching of epithelial cells induces proliferation by activating ERK1/2 and cyclin B transcription, pushing them from early G2 phase towards mitosis [187]. The homeostatic sensor, Piezo1, mediates this effect. Piezo1 is a mechanosensory ion channel that can also drive the up-regulation of c-Jun and endothelin-1, stabilize Hif1a, and drive the proinflammatory response in myeloid cells in response to cyclical hydrostatic pressure [188]. Piezo1 is unique in its ability to detect both stretch and mechanical crowding, forming inactive cytoplasmic aggregates in the latter state.

In addition to SRF and Piezo1, cell shape and mechanical tension transmitted through cell-matrix connections and intercellular junctions exert transcription control through the Hippo pathway. Among the gene regulatory mechanisms, the role of the Hippo/YAP pathway in mechanotransduction is the best characterized. YAP and TAZ are transcriptional regulators frequently activated in human cancers. YAP and TAZ may initiate tumorigenic events that manifest in solid tumors by inducing cancer stem cell proliferation and promoting metastasis and chemo-resistance [189]. Mechanoresponsive factors sense and respond to physical stimuli, such as stretching or increased cell density (crowding), and utilize integrin adhesion and the actin cytoskeleton to induce YAP/TAZ nuclear translocation and the activation of target genes promoting cell proliferation. Mechanical forces activate yorkie (yki), the Drosophila homolog of YAP, in response to stretching of the cell’s apical region. Yki accumulation at the cell cortex in the apical junctional domain results in activation of myosin through a myosin regulatory light chain kinase [190]. Furthermore, changes in F-actin distribution alter YAP/TAZ activity via Rho GTPases, which transmit signals from cell-cell and cell-ECM complexes at the cell surface [191]. Studies in cultured cells have also identified G-protein-coupled receptors (GPCRs) and their agonists as regulators of Hippo signaling [192]. However, different G proteins may have opposing effects on Hippo signaling, with Gα11, Gα12, Gα13, Gαi, Gαo, and Gαq activating and Gαs inhibiting YAP/TAZ [192].

Hippo activity may be affected by increased cell mechanics through direct genomic interactions. Specifically, the tumor suppressor ARID1A operates as an inhibitor of the YAP and TAZ proteins. ARID1A is a prominent component of the chromatin remodeling complex SWI/SNF, which is frequently mutated in various forms of cancers. Loss of ARID1A may represent an obvious pathomechanism for carcinogenesis via de-repression of the pro-oncogenic coactivators, YAP and TAZ. However, a new study has shown that cellular mechanotransduction regulates the association between ARID1A-SWI/SNF and YAP/TAZ. Cells exposed to low mechanical stress experience inhibitory interaction of ARID1A-SWI/SNF and YAP/TAZ, in which YAP/TAZ can freely signal through its DNA-binding platform TEAD. High mechanical stress causes nuclear F-actin to bind to ARID1A-SWI/SNF and prevent the formation of the ARID1A-SWI/SNF-YAP/TAZ complex, thereby enabling an association between TEAD and YAP/TAZ [193].

In search of new druggable factors governing stemness and organ size, alternative avenues for the activation of the Hippo-YAP pathway were identified in genetic screens. In addition to suppressing mTORC1 activity through the phosphorylation of the AMP-activated kinase, LKB1 modulates both microtubule affinity-regulating kinases and Hippo kinases, linking the tumor-suppressive capabilities of LKB1 to YAP activation [194].

Therapeutic opportunities may be presented by modifiers of actin and Rho GTPases, such as gelsolin and cofilin, to inhibit harmful activation of YAP/TAZ leading to enhanced cell proliferation in response to mechanical cues from an abnormal microenvironment. Furthermore, drugs that degrade matrix components and reduce fibrosis may prove beneficial. The angiotensin receptor I blocker, losartan, can reduce both collagen I and hyaluronic acid, as well as inhibit TGF-β action [195].

## 8. Mechanotransduction in the Gut

The gastrointestinal (GI) tract is a mechanically active system; all cells in the GI tract are subject to constant movement. Cells in the GI tract need to sense and/or respond to mechanical signals to perform their physiological function. Thus, mechanotransduction in the GI tract is crucial for normal physiological function, and defects in mechanotransduction lead to a variety of GI pathologies, including chronic constipation, visceral hypersensitivity, irritable bowel syndrome (IBS), and colon cancer [196,197,198]. Mechanotransduction affects gastrointestinal function from the system level to the cellular level. Examples of mechanotransduction include stretch-induced relaxation of the esophageal sphincter and the colon; at the cellular level, increased stretch modulates P21-activated kinase signaling resulting in altered myosin light chain phosphorylation and, consequently, changes in intestinal smooth muscle cell contractility [199,200]. Dysregulation of mechanotransduction contributes significantly to pathology in the gut, ranging from the development of ileus to cancer [201,202]. Thus, understanding mechanotransduction in the gut is crucial for developing successful strategies to treat GI motility disorders and pathologies. Mechanotransduction has been demonstrated in a number of different cell types in the GI tract, including enteric neurons, interstitial cells of Cajal (ICCs), and smooth muscle cells.

### 8.1. Enteric Neurons

The enteric nervous system (ENS) plays an important role in mechanotransduction in the gut. The GI tract is the only organ with an independent nervous system, highlighting the importance of the ENS in coordinating GI motility, secretions, and absorption. The ENS consists of the myenteric plexuses between the two muscle layers in the gastrointestinal wall and the submucosal plexuses. Sensory neurons within the ENS can sense mechanical cues and respond with action potentials [203,204]. The activation of complex ascending and descending pathways in response to stretch, resulting in peristalsis, is an example of the motility patterns induced by mechanical signals in the gut [205]. Shear stress does not appear to play a major role in mechanotransduction in the ENS, while compressive stress plays an important role. Mechanosensitive neurons adapt to compression at different rates. Ion channels play a significant role in the mechanosensitivity of enteric neurons. For example, BK channels are directly mechanosensitive, as discussed above [92,125]. In patch-clamp experiments, membrane stretch comparable to intestinal diameter changes under physiological conditions resulted in prolonged BK channel opening time [206]. Interestingly, mechanical deformation of neuronal processes evokes action potentials in the soma while deformation of the soma body inhibits action potentials [205]. Stretching of S-neurons from the myenteric plexus evoke action potentials, even in the presence of muscle paralytics [207].

### 8.2. Intersitital Cells of Cajal

ICCs are the pacemaker cells of the GI tract. A network of ICCs is located between the two muscle layers of the GI tract and initiates the slow waves (also known as the basic electrical rhythm), which set the pace for GI contractions. The ICCs are in close contact with both enteric neurons and smooth muscle cells. Stretching of gastric muscle induces an increase in the slow-wave rhythm, indicating that ICCs are stretch sensitive [208]. A tetrodotoxin-insensitive voltage-dependent Na^+^ channel appears to be responsible for stretch activation of ICCs [209].

### 8.3. Smooth Muscle Cells

The myogenic response of GI smooth muscle refers to the response of smooth muscle to mechanical forces independent of innervation. The myogenic response is intrinsic to many parts of the GI tract. In the stomach through the colon, slow waves (initiated by ICCs) change the membrane potential of smooth muscle cells; if the membrane potential reaches a threshold, calcium enters smooth muscle cells and triggers a contraction. Stretch can induce smooth muscle contraction in the absence of neuronal influence, indicating that smooth muscle cells are mechanosensitive [205]. A variety of mechanical cues, including shear stress, intracellular pressure, or membrane stretch, induces an influx of Ca^2+^, which likely involves L-type calcium channels [210,211]. L-type calcium channels respond to shear stress and osmotic stress, and these responses are dependent on cell membrane stretching, not cytoskeletal changes [212,213]. TRP and TREK channels may also be involved in mechanotransduction in smooth muscle cells [77,205]. TREK-1 and TRP channels are expressed in gastric and colonic smooth muscle cells [214,215]. Deletion of TRPC4 and TRPC6 results in impaired intestinal motility [216]. BK channels are expressed in colonic smooth muscle and are involved in stretch-induced relaxation of colonic smooth muscle [217]. Blocking BK channels attenuates the relaxation of colonic smooth muscle in response to stretch [217].

In addition to contractile activity, mechanical stretch can induce changes in transcription and intracellular signaling. Shi et al. showed that mechanical stretch in an obstructive bowel disease model induced expression of cyclooxygenase-2 in colonic smooth muscle cells [218]; the induction of COX-2 depended on stretch-induced ion channels and integrin signaling [219]. Intestinal edema, which frequently develops during trauma resuscitation, induces intestinal wall swelling leading to increased stretching of intestinal smooth muscle cells [220]. Stretching of intestinal smooth muscle cells to mimic edema development induces decreased myosin light chain phosphorylation via increased p21-activated kinase activity [199,200].

### 8.4. Other Cell Types

A number of different endocrine cells reside in the GI mucosa, and many of these cells are mechanosensitive. Mechanical stimulation of the intestinal mucosa induces the release of serotonin from enterochromaffin cells, which affects the ENS [221]. TRPA1 channels may be involved in mechanotransduction in enterochromaffin cells [205]. A wide variety of immune cells reside in the gastrointestinal tract, including resident macrophages in the intestinal wall. These cells may also respond to stretch and release inflammatory mediators [201]. Macrophages also respond to pressure by increasing phagocytosis and cytokine release, possibly through focal adhesion kinase and extracellular signal-related kinase inhibition [222]. Epithelial cells and vascular endothelial cells are also responsive to mechanical forces [223].

## 9. Conclusions

Virtually every cell responds to intrinsic and extrinsic mechanical cues. Most of these mechanical signals are sensed and transmitted directly at the plasma membrane or the interface between the cytoskeleton and the plasma membrane at cell-cell and cell-membrane adhesions. These adhesions link cell signaling to the surrounding environment and changes in the mechanical characteristics of the environment are transduced to intracellular signals. Mechanotransduction plays a significant role in both physiological and pathological functions. In this review, we discussed the contribution of mechanotransduction to normal physiological functions using the gut as an example and the contribution of mechanotransduction to pathological conditions, using cancer development as an example. There are still many unanswered questions about how mechanical signals are sensed by proteins and how these signals are transmitted into and through the cell. Understanding mechanotransduction in health and disease will facilitate the identification of new drug targets.

## Figures and Tables

**Table 1:** The diverse roles of mechanosensitive mammalian ion channels.

Ion Channel Family	Mechanosensitive Forms	Examples of Physiological Relevance	References
ENaC	ASIC1	Gut mechanosensation	[60,61]
	ASIC2	Arterial baroreceptor reflex; cutaneous touch	[62,63]
	ASIC3	Gut mechanosensation; presure induced vasodilation; nociception	[60,61,64,65]
	βENaC	Myogenic vasoconstriciton	[66]
	γENaC	Myogenic vasoconstriciton	[66]
Piezo	Piezo 1	Vascular developmental/shear stress response; touch sensation; red blood cell function; bone growth; sensing bladder distension	[67,68,69,70,71,72]
	Piezo 2	Touch sensation; enterochromaffin response to mechanical signals	[73,74,75]
TREK	TREK1	Pain perception; mechanosensation in the gut; vasodilation	[76,77,78]
	TREK2	Pain perception	[79]
TRP	TRPA1	Touch sensation, pain perception	[80,81,82]
	TRPP1	Response to flow in renal epithelium; endothelial/epithelial cilia function	[83,84,85]
	TRPC6	*Vascular smooth muscle contractiity (conflicting results)*	[86,87,88]
	TRPP2	Response to flow in renal epithelium	[83]
	TRPV4	Sensing weight load during bone development; micturition reflex; pressure sensing	[89,90,91]
BK	BK_Ca_	Mechanosensation in the gut; flow-induced K^+^ secretion in nephrons	[92,93]

## Data Availability

Not applicable.
